# Identifying User Interaction Patterns in E-Textbooks

**DOI:** 10.1155/2015/981520

**Published:** 2015-10-29

**Authors:** Santeri Saarinen, Tomi Heimonen, Markku Turunen, Mirjamaija Mikkilä-Erdmann, Roope Raisamo, Norbert Erdmann, Sari Yrjänäinen, Tuuli Keskinen

**Affiliations:** ^1^TAUCHI, School of Information Sciences, University of Tampere, Kanslerinrinne 1, 33014 Tampere, Finland; ^2^Department of Teacher Education, University of Turku, Assistentinkatu 5, 20500 Turku, Finland

## Abstract

We introduce a new architecture for e-textbooks which contains two navigational aids: an index and a concept map. We report results from an evaluation in a university setting with 99 students. The interaction sequences of the users were captured during the user study. We found several clusters of user interaction types in our data. Three separate user types were identified based on the interaction sequences: passive user, term clicker, and concept map user. We also discovered that with the concept map interface users started to interact with the application significantly sooner than with the index interface. Overall, our findings suggest that analysis of interaction patterns allows deeper insights into the use of e-textbooks than is afforded by summative evaluation.

## 1. Introduction

Recently, electronic textbooks are becoming popular in schools; however, there are a lot of differences in the ways how the electronic material is used, and no common guidelines to use e-books exist. To effectively use digital material in schools, we need to identify how they can enhance learning situations most efficiently. Electronic texts have many benefits; for example, Murray and Perez [1] mention “widespread accessibility, interactivity, increased visual appeal and dynamic linking to supplemental materials.” However, previous research suggests that resulting learning outcomes from using electronic material often seem not to differ from those achieved using printed textbooks [1, 2]. Thus, traditional linear text in electronic form does not bring significant benefits by itself. One possible solution to this problem is to provide advanced navigational aids or additional representations of the e-text, as we have done in our previous research [3, 4]. The goal of our study reported here was to find out how different navigational aids affect students' learning outcomes and how their reading patterns differ when using the aids. We used concept maps [5, 6] as the main navigational aid. Our solution is novel in the e-learning field, since previous research has concentrated on the use of standalone concept maps, for example, [7, 8], or their use as an assessment tool [9]. Overall, previous research on the use and efficacy of concept maps as part of e-texts in higher education is very sparse [3]. Our research offers information on how concept maps used in combination with e-text can affect learning situations on mobile devices and how these kinds of services should be evaluated.

In universities, the main method of science teaching is often still textbooks, in which the information is organized by topic, to be read and understood in a linear fashion [2, 10]. However, textbooks are often not organized well enough or do not explain the phenomena coherently and rarely contain questions that require students to provide comprehensive explanations [11, 12]. E-material is often created based on these existing textbooks, and thus the content is mostly static. Furthermore, interaction capabilities are often fairly basic, compared to the latest new solutions offered by the different interaction techniques on (touch-enabled) laptops and mobile devices. Students are skilled in nonlinear navigation and dynamic interaction, which they are actively pursuing everywhere else, except in the classroom. Because of this, there is a clear need to develop e-learning material to become more interactive and dynamic, which allows the students to utilize the skills they have developed during their free time.

Vassiliou and Rowley define e-book as “a digital object with textual and/or other content” and which commonly contains “in-use features, hypertext links, bookmarks, annotations, highlights, multimedia objects and interactive tools” [13]. Earlier research on e-textbooks has mainly focused on reading speed and comprehension of individuals using e-material, instead of trying to identify methods which enable learners to interact more efficiently with the material. Also, the number of studies which discuss e-textbooks as a mobile learning tool is fairly low [1]. In addition, multimodal learning technologies have not been widely taken into use in schools yet, even if there has been a lot of promise in related research in school contexts [14, 15]. However, these multimodal solutions often required some additional technology or devices. With the latest developments in mobile technology, a simple tablet or mobile phone could offer just as a suitable platform for learning software as the larger and more specialized computers. And on the other hand, mobile technology is so widely available these days that taking it into use in a classroom does not require large investments from the universities.

In our research we studied students' interactions with e-textbook while utilizing different navigational aids. The main tool we chose was concept maps, which are graphical tools for organizing and representing knowledge. Their main elements are named concepts (usually enclosed in circles or boxes of some type) and relationships, which link two of these concepts to create a meaningful statement or proposition in the form of a diagram [5]. One of concept maps' main applications in education has been to support writing and reading activities. There are three different ways of doing this: building a concept map, editing previously built concept map, and studying an existing concept map [16]. Previous research has shown that each of these activities can improve the students' understanding of the provided material [17, 18]. Nesbit and Adesope have concluded that students' understanding of studied material improves, because concept maps remove redundant information and help with the understanding of relations between concepts because they are often colocated on the map [19]. According to Hauser et al. [18], studying with a ready-made concept map enhances learning most effectively compared to the two other alternative ways of using concept maps. Concept maps have also been shown to boost students' feeling of achievement and reduce their cognitive load while studying when they were embedded in an educational game [20]. These results motivated us to select concept map as our main navigational aid and especially concentrate on the ready-made map as an embedded aid in the e-textbook.

In our analysis of user data, we could identify three distinct interaction patterns, one of which only appeared with students using the concept map as a navigational aid. Our results also show that the use of concept map also significantly decreased the time it took for the users to start interacting with the e-textbook application. Based on our results, we can conclude that using visual aids in e-textbooks offers potential benefits to the user experience and that, with proper interaction analysis, we can identify different usage patterns which could allow us to automatically customize the learning software to better fit the user's reading style and offer useful information to the teacher during the use of the material.

In the remainder of the paper, we first present our study and the application developed and then describe the user test we carried out and present the data and results from our analysis. Finally, we will discuss the results and possibilities for future research and conclude with our main findings.

## 2. Materials and Methods

In this chapter, we will first present Eager, the e-textbook application we created for the purposes of our study. Then, we will describe the evaluation study conducted with the application. Finally, we discuss the analysis of interaction sequences and patterns in detail. For this research, biology for teacher education students was selected as the learning context, because earlier research has shown that students in teacher education have several misconceptions about biology [21]. We created suitable e-material in cooperation with teachers familiar with the subject, concentrating on changing common misconceptions relating to the phenomena studied.

### 2.1. Overview of the Eager Application

We have developed a web service called Eager to test our interface designs for e-textbooks. The Eager service supports different platforms and device configurations and can be used on most mobile phones and tablets. The end user application is an interactive webpage, which includes the containers for data and the user interface with the main functionalities of the application. The data (actual content of the e-textbook and all accompanying material) is saved in XML-format on the server running the service, which can be accessed through HTTPS calls. Based on which part of the application is being used, data for that section is downloaded from the server and parsed dynamically. This is done to avoid long waiting periods during the use because of the need to download large amount of files to the user's device. This approach allows us to expand the scope of the application easily and make it scalable as the amount of data contained in the application does not create any restrictions. The graphical parts of the application are using D3.js library [22], which provides tools for visualizing data and the possibility to update these visualizations dynamically if the data changes while the application is running. In this phase of the research, we used static data, so the visualizations were similar for all users.

For the user study reported here, Eager was optimized for Safari web browser on iPad Air tablet with 9′7′′ screen and 2048 × 1536 resolution on iOS version 7.0.4. These specifications were selected because of practical reasons. The university where we held the evaluation sessions had acquired a large number of these tablets for students' use, and we were offered the opportunity to use them in our study.

### 2.2. Eager User Interface

**Figure 1:**
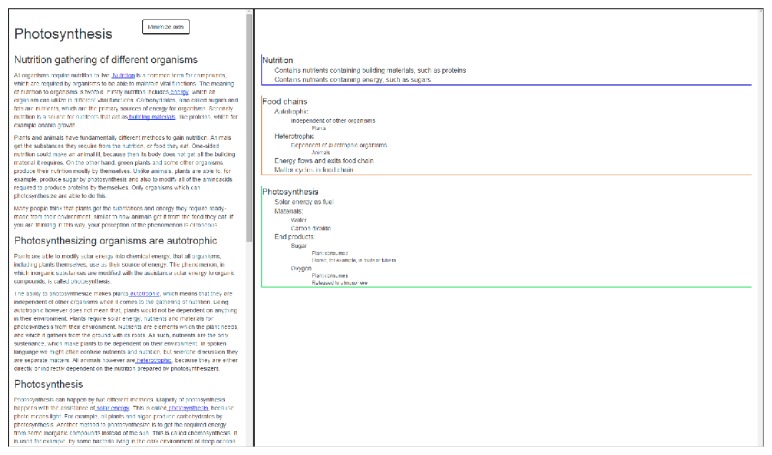
The index version of the Eager application.

**Figure 2:**
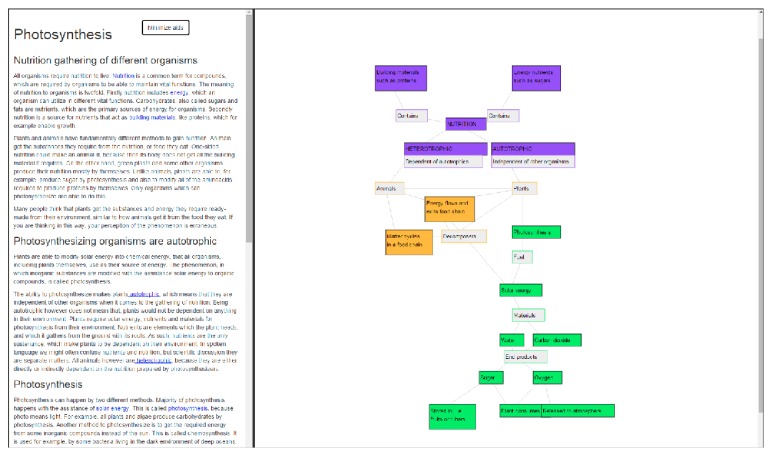
The concept map version of the Eager application.

The Eager user interface consists of two separate but linked parts. Left side of the screen contains the traditional e-text, while the right side of the screen has the navigational aids linked to the e-text. We created two different versions of the applications with different aids. We selected* concept map* as our main navigational aid, because it has been shown to be an effective tool for learning in the natural science context [20]. As the baseline navigational aid, we selected* index* (i.e., table of contents), which is common in most e-textbooks. We altered the normal functionality of index to be more comparable with the concept map and to offer the same information. In practice, this means that the index is not organized linearly but in a hierarchical order based on the relationships of the terms. This hierarchy appears within the three separate blocks, visualized by the different indents of the terms. Figures 1 and 2 show both versions of the application. All of the texts and interface features in these figures are in Finnish, because that was the language used during the evaluation.

Figure 1 shows the version of the Eager application, which has index as a navigational aid. The important terms, definitions, and concepts are presented in a hierarchical index in this version. They are separated to different blocks under overarching terms, such as nutrition, food chains, and photosynthesis. Within these blocks, the different terms are organized in a hierarchical system. Whenever a user clicks a term within the e-text, that term is highlighted in the index. Simultaneously, the whole block of concepts to which the term belongs is bolded to emphasize the relations of the term and its position in a hierarchy.

Figure 2 shows the version where the navigational aid is a concept map. In this version, the terms are laid down in a graph to better emphasize their connections instead of their hierarchy. The concept map is color-coded similarly to the index version, to indicate the larger wholes to which different terms belong. The outlook of nodes also differs based on the type of information they contain. Finally, the nodes are clustered or placed to certain positions to better describe their relations to other nodes. The teacher can freely edit the concept map while creating the learning material and define the above attributes.

When a user clicks a term in this version, the corresponding node is highlighted with a slight color change and bolding of the text. Also the screen is scrolled automatically in a way that places the selected node in the middle. This is done because the concept map could be too large for the screen and all parts of it are not always visible, especially when the e-text side takes up a large part of the screen space. Additionally, the group of nodes to which the term belongs is highlighted, similarly to the index version. The linking of terms also works in the opposite direction. Whenever a user clicks a node in the map, the corresponding word in the e-text is highlighted and the view is scrolled accordingly to the first appearance of this term in the text.

The e-text side of the application is similar in both versions. In the text, the main concepts and terms of the studied subject are marked with blue color and underlining to mark them as links. This kind of markup is common in many websites and applications and thus should be clear to the users. The teacher can select which terms should be highlighted, and they have their special markup in the XML. By clicking these terms, the user activates the other side of the application, which then offers the students more information about the connections of this particular term.

Common to both versions of the application is also the way they handle screen manipulation. The screen is locked to horizontal view. By default, the e-text side takes up two-thirds of the screen, while the navigational aid fills the remaining third of the screen. The user can change this with a single click of a button to increase the size of the navigational aid, or vice versa, whenever the user so chooses. It is, however, not possible to put either part of the application to complete full screen mode, and thus both sides are visible all the time.

### 2.3. User Evaluation of the Eager Application

The Eager application was evaluated during spring 2014. Altogether 99 native Finnish-speaking second-year undergraduate students from the Faculty of Teacher Education of the University of Turku in Finland participated in the evaluation sessions. 23% of the participants were male and 77% were female, and they were between 21 and 42 years old (median age 26). The evaluations were conducted during four sessions, during students' regular class times, and with their regular teacher supervising the evaluation. The first two groups tested the application on the first day and the other two groups on the following day. Additionally, students who were not able to attend these days took part in separate sessions organized a week later. The participants were randomly divided into two groups, where half of the students used the Eager with index as the navigational aid (*N* = 49) and half used concept map as the navigational aid (*N* = 50). All four evaluation sessions were identical, roughly equal in size, and containing an even number of randomized users for both versions of the application. The evaluation was divided to these sessions based on students' available schedule and the evaluators' ability to handle a certain amount of people taking part in the evaluation at the same time.

A single evaluation session lasted for 90 minutes, during which the participants had to complete the three parts of the test: the pretest questionnaires, the use of the application, and the posttest questionnaires. The total time was limited, but the students were free to divide the total time as they wanted between the parts of the test. All sessions were completed ahead of the planned schedule, between 60 and 70 minutes, so we can safely assume that participants were able to spend sufficient time with the application and related questionnaires during the evaluation. For the evaluation, we chose photosynthesis as topic of the material. It is one of the most important and also most challenging topics in biology. 91% of the students had completed a basic course on biology in their university studies. The course concentrated on the phenomena studied at elementary school level, and thus the large majority of the participants were at least cursorily familiar with the subject studied during the evaluation.

During the evaluation we used pre- and posttest design [21, 23]. In the pretest part, the participants filled in background information and answered open-ended questions about photosynthesis and related concepts. These included both factual questions and generative questions. After answering these, the students proceeded to use the Eager application and study the material at their own pace. The material focused on self-sufficient organisms, the nourishment supply of plants, the flow of energy in the food chain, and the significance of photosynthesis to life on Earth. The e-learning material was designed in such a way that the paragraphs point out typical misconceptions concerning the phenomena. The text also discusses concept pairs and their relationships, such as energy versus matter or nutrients versus nourishment. These concepts were chosen based on previous literature, which has shown that both adults and children have difficulties understanding them [11, 12, 24].

After studying with the Eager application, the participants answered the same open-ended questions again without the text. They were allowed to alter their earlier answers if they felt that they had gained new information while studying. Finally, the participants answered a user experience questionnaire, created based on the SUXES-method [25]. With the questionnaire we obtained information about the students' experiences on the use of the application. These experiences were collected with a questionnaire consisting of statements answered on a 7-step Likert scale. The participants also answered some open-ended questions which helped us to understand how they wished to use digital learning applications and guided our future iterations of the software. To evaluate participants' learning outcomes, we scored the pre- and posttests. Answers to the open-ended questions were scored as incorrect, partial, or correct. The responses were given points based on a rubric modified from earlier research [6]. The scores given for pre- and posttests will be used as dependent variable in the variance analysis conducted.

On top of the subjective data collected from the participants, we collected anonymous data from the use of the application with a manual instrumentation approach [26]. The particular type of observational data we are interested in is the logging of user interface events, as these can be inexpensively collected during the use of the application. Collecting all action events from the use allowed us to analyze the usage of the application and recreate the users' interactions with the application. In future iterations of the application, we are going to utilize a dedicated instrumentation framework which allows us to define the data we want to collect more effectively.

### 2.4. Interaction Analysis

One of the ways to analyze the use of an interactive application is to identify the sequences of interactions and behaviors by adopting an exploratory sequential data analysis (ESDA) approach [27], which helps to gain a deeper level of understanding how the interaction takes place [28]. While traditional ESDA methods are often grounded on a top-down approach whereby the manipulation of the observational data is guided by research questions, another approach is to proceed bottom-up, that is, collecting a mix of interaction data to support a wide variety of investigations without a predefined thesis in order to identify interesting behavioral patterns [29]. This is the approach adopted in this study.

There are several challenges in analyzing log data based on interface events, such as taking into account the relationship between low and high level events, the proper interpretation of context (which is often lacking in individual events), and addressing multiple levels of abstraction, from physical events to task and problem related interactions [30]. It should be kept in mind, however, that users' logged interactions and movements alone are not enough when studying their intentions, expectations, and experiences. In addition to system events and interactions, contextual information is needed to identify the reasons behind the detected behaviors [28]. As an example, contextual experience sampling methods have been used to study mobile application use by triggering the collection of user feedback when events of interest take place [31, 32]. Alternatively, users can be asked to reflect their behaviors after the fact, using, for example, questionnaires [33]. Overall, mixed methods approaches combining quantitative analysis of system use with qualitative feedback are considered an effective solution in studying interaction behaviors [34]. Our study utilized a mixed methods approach, whereby the interaction log data was supplemented with subjective feedback and pre-/posttest learning assessment.

Captured interaction log data typically needs some form of transformation to be suitable for automatic analytical processing, for example, by selecting items to analyze, abstracting them to higher-level events, or creating new, more abstracted logs through recording [30]. Hilbert and Redmiles [30] identify approaches for analyzing interaction sequences, including sequence detection, comparison, and characterization. Sequence detection means the extracting of patterns of interest from log data that are known in advance, for example, based on a research hypothesis. Sequence comparison pertains to making comparisons between patterns, such as those from different user groups. Finally, sequence characterization is the process of extracting salient patterns in the interaction data, for example, in the form of a process model of interaction. In practice, several challenges exist, when applying data mining algorithms to the analysis of interaction data. For example, the patterns can be redundant and difficult to understand, care should be taken to avoid biasing the analysis when grouping patterns, and there is a need to experiment with different criterion functions for clustering to fully understand the relationships among patterns [35].

While sequence detection can answer research questions related to expected patterns, for example, by utilizing predefined task models, the advantage of sequence characterization is that the patterns emerge from the data and are not based on existing models, which allows the discovery of potentially novel insights. As an example, Mankowski et al. [36] developed a technique that automatically finds representative sequences of behaviors without the need for prior models. Moreover, effective visualizations are critical for understanding the captured behaviors and identified patterns. Examples from the domain of game analytics [37, 38] suggest that interactive spatiotemporal visualizations that allow data aggregation, filtering, and clustering of similar behaviors can be helpful to understand player behavior in games.

One of the potential benefits of interaction sequence analysis is that it can also be used to detect different user characteristics, such as skill level. This information can then be used online either to adapt the application or to predict future behavior. For example, Hurst et al. [39] implemented a classifier that could detect novice and skilled use of an image-editing program based on interface events. Huang et al. [40] analyzed how patterns of play are related to skill acquisition in a first-person shooting game. Their model was able to explain how factors such as play intensity and breaks in early games may affect players' skill level. Although we did not attempt online detection of the participants' understanding of the photosynthesis phenomena in this study, attempting to relate pre- and posttest scores and interactions is an interesting avenue for our future work. For example, we could try to predict participants' understanding from their interactions during first three minutes of reading and see if this correlates with the actual score differences. This kind of prediction would allow us to customize the later text based on the early actions of users, for example, by offering more or less links to navigational aids.

## 3. Results and Discussion

In this chapter, we will describe the results obtained from our evaluation study of the Eager application. We will first present the results of our interaction sequence analysis. After this, we will briefly describe the results of the user experience questionnaire and investigate its relationship with participants' learning outcomes. Finally, we will discuss the implications of these results and describe our future research agenda.

### 3.1. Interaction Sequence Analysis

**Figure 3:**
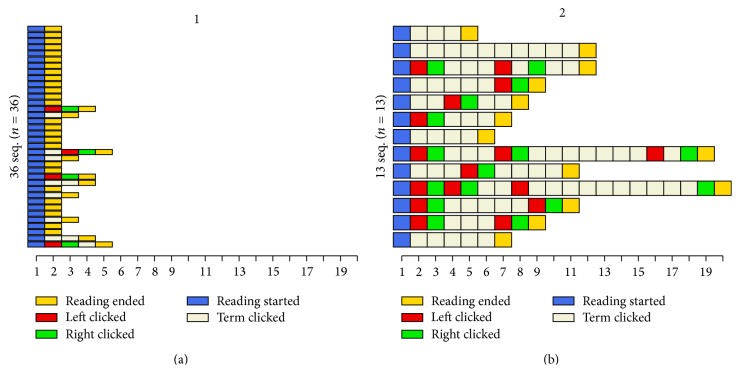
Cluster solution generated by TraMineR for the index version. “Passive users” (a) and “term clickers” (b).

**Figure 4:**
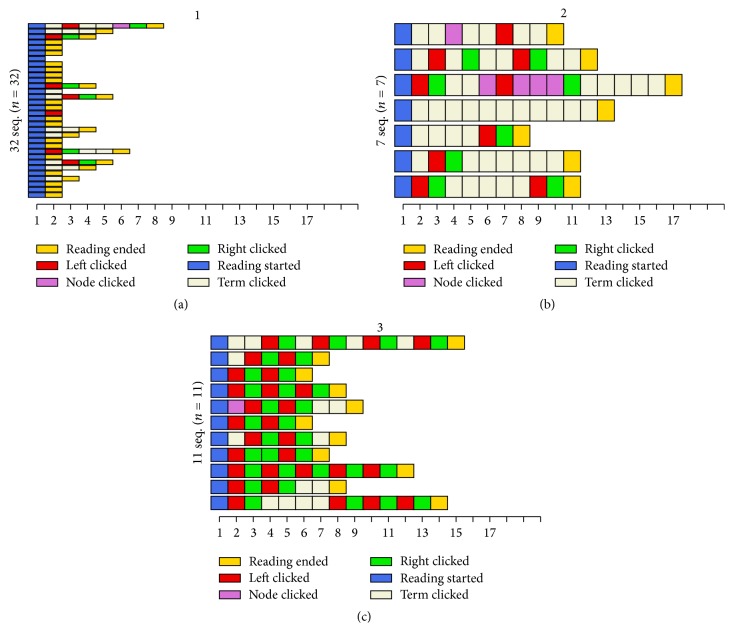
Cluster solution generated by TraMineR for the concept map version. “Passive users” (a), “concept map users” (c), and “term clickers” (b).

Users' interaction sequences with the application were extracted from log files. This was done by python scripts which gathered the data from the separate log files and saved them in a CSV file which could then be analyzed. The sequences consisted of three user interaction events (minimize text, minimize visual aids, and select term) and two markers signifying the start and end of the reading session. For the concept map version, an additional interaction event was added for node selection. In the following cluster solution, Figures 3 and 4, these interactions are marked as follows: markers for the start and end of the session are called “reading started” and “reading ended,” minimizing text equals “left clicked,” minimizing visual aids equals “right clicked,” and term selection is marked as “term clicked.” These interactions were selected to be tracked because aside from scrolling or rotating the screen, they are the only interactions available while the user is studying the material, and they allow us to track the whole sequence of actions taken during the use of the application instead of focusing only on certain aspects of interaction. The sequences were analyzed using the statistical computing software R [41] and the packages* TraMineR* [42] and* cluster* [43]. Sequence dissimilarity was calculated using the optimal matching metric with substitution costs based on state transition rates. Ward's method [44] was applied to hierarchical clustering, resulting in a solution with two clusters for the index version (Figure 3) and a solution with three clusters for the concept map version of the interface (Figure 4). In Figures 3 and 4, the *y*-axis describes the number of users and the *x*-axis the number of actions. Each cell is a single action, colored based on the type of action. The users are divided to the different clusters based on the characteristics of their interaction sequences. Cluster solutions that provided* TraMineR* offered a starting point for the analysis. A manual inspection showed that the characteristics of some users would better fit the main characteristics of another cluster, and these users were moved manually to the correct category. Because of this, the numbers provided in Figures 3 and 4 do not equal the percentages below. Final amounts of users in each category were 31 users in cluster 1 and 18 users in cluster 2, for index version, and 23 users in cluster 1, 12 users in cluster 2, and 15 users in cluster 3 for concept map version.

Qualitative visual inspection of the clusters revealed salient differences in the participants' behaviors with the interface. We named the clusters based on these differences in the style of interaction. With both interfaces, a large proportion of participants belonged to the “passive user” category (63% with the index version and 46% with the concept map version) and consequently did not use, or made minimal use (<2 interactions) of, the interface features and only concentrated on reading the text. The second category of participants with the index interface can be classified as intermediate to active users of interface features. Although selecting terms was the most frequent type of interaction, 72% of the participants in the second category also made use of the functions to adjust the screen space division between the text and index at least once.

The participants in the concept map version differ in their interaction profiles and we could identify two distinct groups of users in addition to the passive users: “term clickers” and “concept map users” (Figure 4). The former group (24% of participants in the concept map condition) primarily utilized the terms and nodes as a means of interacting with the concept map. The latter group (30%) primarily relied on readjusting the screen real estate provided for the text and concept map to take turns studying them both in more detail.

**Figure 5:**
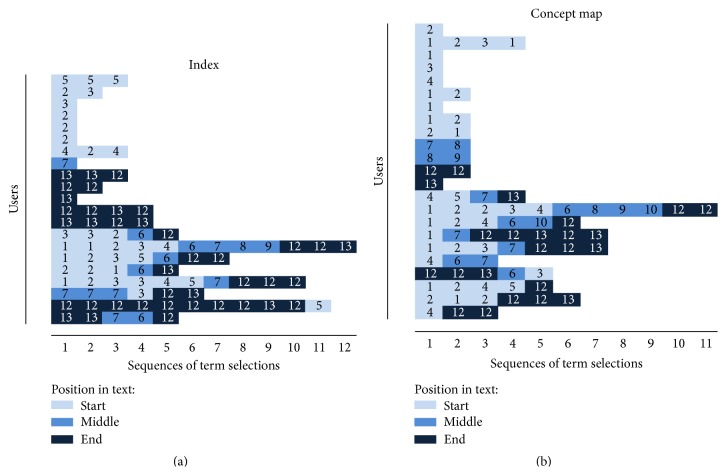
Term selection sequences for both UI versions.

In addition to the overall interaction patterns, we investigated the participants' selections of terms in the text in order to understand the relationship between interface use and the content access patterns. We did not combine this with the information gained from the cluster analysis, because dividing the samples into smaller groups would have diminished the frequencies even further, and comparison of these extremely small samples would not have been able to offer meaningful information. We analyzed the term selection sequences with respect to the order of the terms' appearance within the text (1st to 13th). We divided the chapters into three parts and analyzed users' interactions based on which of these three parts the terms were located in. Because the terms were marked only on their first appearance in the text, the text was a bit front-loaded with terms. First part contained five terms (terms 1–5), middle part contained six terms (terms 6–11), and last part contained only two terms (terms 12-13). In both versions, roughly half of the participants selected terms within the text: in the index version 43% of the participants and in the concept map version 46% of participants did so. We analyzed the term selection patterns based on the locations of terms within the text combined with the order the users selected them. We identified six different term selection behaviors (Figure 5). Figure 5 shows the term selection sequences of the users in both interface versions. The *y*-axis shows different users. The *x*-axis describes the amount of term selections. The coloring of the nodes shows which part of the text the term belongs to, and the number describes the actual index of the term in the text. The terms appear in the text in their numbered order (term 1 first, term 13 last). The frequencies of identified behaviors are described in Table 1 by version. Interestingly, the term “oxygen” (11th term) did not receive any selections in either version.

The most prevalent form of term selection in both versions (33% in index version and 35% in concept map version) was to select terms at the beginning of the text only (the five terms in the first part of the text). In both versions, this was followed by selecting terms in the order they appeared in the text (24% and 26%). One-fourth of the participants in the index version selected terms at the end of the text after having read through the text (24%), with nearly half of them also backtracking to the beginning of the text (10%). This behavior was much less prevalent in the concept map version with only 9% of users selecting terms at the end of the text and only 4% backtracking. It was more common for the participants to select terms either in the middle parts of the text (13%) or in the beginning and end of the text (13%).

In order to analyze the interaction with the system quantitatively, we calculated time-based measure* time to first interaction* which attempts to quantify how quickly the user's engagement with the application started. This value was calculated by taking the time between the “reading started” marker, which denotes when user started using the application, and first appearance of any other interaction. The time to first interaction was significantly lower in the concept map version (mean = 135 seconds, median = 18 seconds, and *n* = 31) than in the index version (mean = 261 seconds, median = 77 seconds, and *n* = 23), Mann-Whitney *U* = 243.0, *P* < 0.05.

### 3.2. User Experience Analysis

In addition to an analysis of the interaction metrics in isolation, we examined the relationship of amount of interactions with the perceived experience with the two interfaces. We collected the user experiences with a user experience questionnaire utilizing the SUXES-method [25]. In the questionnaire, the participants answered to statements regarding their experiences with the application. These statements were answered on a 7-step Likert scale. In the answering scale value 1 corresponds to, and was labeled as,* Totally disagree*, 4 corresponds to* Neither agree nor disagree*, and 7 corresponds to* Totally agree*. The median results of the questionnaire are shown in Table 2. For the user experience questionnaire analysis, we used median ratings instead of mean ratings, because, with an ordinal scale, these help us to reduce the effect of singular outlier answers on the scale and give more accurate description of the responses.

The statements appearing in the user experience questionnaire were designed based on the SUXES-method. Statements 1 through 9 relate to the user experience of the application and are derived straight from the SUXES-method. They relate to speed, pleasantness, clearness, error-free use, robustness, learning curve, naturalness, usefulness, and future use of the application or modality evaluated [19]. Statements 10 and 11 relate to the general satisfaction with the application and its perceived effectiveness on assisting with learning. Statements 12 through 17 evaluate similar things but are specifically related to the navigational aids, concept map, and index and their usefulness in understanding the relations between different concepts.

The responses to the user experience questionnaire indicate that the participants rated the application in general very positively, regardless of the interface version (median response ≥ 6). In the questionnaire, the term glossary covers the different navigational aids. Statements about the use of these (12–17) were also rated positively in both interface versions (median response ≥ 5), except in the case of helping the users to identify errors in their thinking (statement 15). This was rated as neutral in both versions, which could suggest that the users felt there was no need to identify errors, as the majority of them were already familiar with the concepts used in the material. One of the reasons for this may be the brevity of the learning material. This could also support the fact that the ability of the glossary to support learning and analytical thinking was rated slightly lower (statements 14–17). It is possible that with a more extensive, unfamiliar content the visualizations would have provided better support. The lower (but still positive) scores related to willingness to utilize the application again (statement 9) could also be explained by this, because the very specific context of the application during the evaluation does not offer a huge value for users after the first time if they feel they have learned enough about the phenomenon. Overall, we did not observe statistically significant differences between the interface conditions.

To find out if there were differences between the identified interaction pattern clusters within each interface version, we conducted a one-way ANOVA with multiple comparisons using the Bonferroni correction. We found no meaningful differences for the index version of the interface. However, in the concept map version, there were significant differences in some questions when comparing with different interaction patterns. For statement 11, the overall satisfaction with the application, *F*(2,47) = 3.35, *P* = 0.043, the passive users (mean 5.74) were less satisfied than the concept map users (mean 6.67), *t*(33) = −2.49, *P* = 0.050. For statement 12, the usefulness of the glossary, *F*(2,45) = 5.38, *P* = 0.008, the term clickers (mean 3.92) felt it was less useful for them than the concept map users (mean 5.93), *t*(25) = −3.27, *P* = 0.006. For statement 13, the annoyance caused by the glossary, *F*(2,45) = 5.28, *P* = 0.009, the term clickers (mean 2.92) were more annoyed than concept map users (mean 1.33), *t*(25) = 3.12, *P* = 0.009.

With respect to the user experience ratings, we observed moderate positive correlation in the concept map version between the number of interactions carried out and the perceived usefulness of the interface, *r*_*s*_ = 0.33, *P* < 0.05, and the application assisting with learning, *r*_*s*_ = 0.30, *P* < 0.05. With the index map version, there was a moderate positive correlation between number of interactions and the degree of perceived annoyance with the content of the index, *r*_*s*_ = 0.32, *P* < 0.05.

We also looked at the relationship of interaction metrics and learning outcomes, to find out whether different ways to use the application would affect the outcomes. As a measure of learning outcome, we computed the difference between participants' pretest and posttest assessment scores. There were no significant correlations between the number of interactions and test score change between the pretest and posttest assessment. When comparing the identified interaction patterns with the scores, we found no significant differences between the pattern clusters and score change. However, when comparing with only the posttest score, we did find significant differences in the concept map version of the interface, *F*(2,47) = 6.58, *P* = 0.003. Term clickers (mean 23.83) performed on average better than both nonusers (mean 18.39), *t*(33) = 3.51, *P* = 0.003, and concept map users (mean 19.03), *t*(25) = 2.85, *P* = 0.020. No significant differences were found when comparing index version's clusters with posttest score.

## 4. Discussion

As our results show, the use of concept map as the navigational aid decreased the time it took for participants to start interacting with the application. Users with the concept map version also used the application slightly longer, as reported in our earlier work [4]. This group also had a higher percentage of users who interacted with the e-textbook. The group of users who used the concept map version of the interface, and who, based on their interaction pattern, were categorized as concept map users, rated the usefulness of the application and its assistance in learning higher than the average user. These results could point to the conclusion that using tools such as concept maps, or other visual aids in combination with e-texts, would improve the perceived usefulness of an e-learning application. Based on this, even though short term usage did not seem to have immediate effects on learning outcomes in the case of this rather short e-text material, the increased interest in learning by the students, combined with the use of new technologies, could with more experience lead to better results when longer e-material is used.

In this kind of analysis of user interaction, the use of mixed methods offers valuable and supplementary data. This was also noticeable in our results. By comparing the interaction pattern data with the user experience, we could identify certain types of users that experienced the application differently. For example, the passive users were less satisfied with the application. This points to the direction that we should try to engage more users to interact with the application if we wanted to increase their overall satisfaction.

By analyzing the interaction patterns alone, we could already identify several different types of users, namely, passive users, term clickers, and concept map users. Combining this kind of analysis in the future with the possibility to track the actual focused part of text (e.g., by using suitable user interface techniques and/or gaze tracking), we could possibly identify different reader types straight from the data. For example, we would be able to see whether the students first skimmed through the text and then returned to the beginning, or if they read the whole text linearly. By analyzing how long the users are using each part of the interface, we could also identify whether they prefer learning more by reading the text or by researching the concept maps, for example. This would allow us to identify different types of users from a fairly small amount of data and offer them more customized content that fits their learning habits even during a single course. For example, if we could identify the preferred reading style of a user during the first few lectures, we could change the available content by, for example, showing more links to images, if a user seems to prefer looking at them.

Based on the results of our study, and the feedback received from the participants, we have iterated the user interface and added new functionalities to the next version of the e-textbook. This version will be tested with another group of university students. We will also widen the scope of our research and run evaluations with high school students, who are not as familiar with the subject as the university students are.

We have also improved our logging methods, to increase the precision of tracking users' actions. In the latest version, we aim especially to be able to track which part of the text the user is reading based on page scrolling and the position of the text on the screen. This allows us to identify which chapter a student is currently reading without the need for eye-tracking and thus allows us to see which parts are quickly skimmed through and which parts cause problems for students. This is done by comparing the location of top and bottom parts of the screen and the relative coordinates of each paragraph. Based on this comparison, we can identify the paragraphs which are shown on the screen currently and how they are positioned. Combined with timestamps of each interaction, we can see how long the users spend reading each part of the text. This may enable identifying learning problems from interaction logs (e.g., similarly to using backtracking actions to identify usability problems in created oriented applications [32]).

## 5. Conclusions

In this paper, we investigated e-textbook navigation aids. We compared two interfaces with different navigation aids with 99 second-year undergraduate university students. Our results show that using a concept map as a navigational aid decreased the time it took for students to start interacting with the application compared to the index version of the interface.

In the concept map interface version, the users who had more interactions with the application also rated the usefulness of the application higher and felt that it assisted their learning more. Similar correlations were not found in the index version. Because of this we can conclude that using navigational aids, such as concept maps, could improve the perceived usefulness of e-text material, since it both encourages the users to start interacting with the application and improves their opinion on its usefulness.

We also identified three different user types from the interaction sequences of the users: passive user, term clicker, and concept map user. Two of these were present in the index condition and all three in the concept map condition. This could mean that visual navigational aids allow for more diverse learning styles, as they seem to encourage different ways of interacting with an application. We will continue to research this in the future iterations of our application. The possibility to identify learning styles purely from the log data would allow us to offer important information to teachers during a course, so they can alter their teaching methods accordingly to support their students, and to customize the learners' e-material with respect to their method of studying.

We have also shown that the use of mixed methods in the evaluation, for example, combining user experience data with interaction data, is effective when analyzing mobile learning applications. This kind of evaluation offers supplementary data, which can be extremely useful when developing those applications. By combining different methods, we can discover information that would not become clear by just using a single method of evaluation. As our results have shown, we gained significant information by using mixed methods and it helped us to understand the data better.

## Figures and Tables

**Table 1:** Frequencies of term selection patterns by interface version. Differences to 100% are due to rounding.

Type	Index	Concept map
Select terms in order of appearance in the text	24% (5)	26% (6)
Select terms at the beginning and end of the text	0% (0)	13% (3)
Select terms at the end of the text	24% (5)	9% (2)
Select terms at the end of the text and backtrack to start	10% (2)	4% (1)
Select terms at the beginning of the text only	33% (7)	35% (8)
Select terms at the middle of the text	5% (1)	13% (3)
None of the above	5% (1)	0% (0)

**Table 2:** Median user experience ratings for the interface versions.

	Index	Concept map
(1) Using the application was fast.	7	7
(2) Using the application was pleasant.	7	7
(3) Using the application was clear.	7	7
(4) Using the application correctly was effortless.	7	7
(5) The application functioned in an error-free manner.	7	7
(6) It was easy to learn to use the application.	7	7
(7) Using the application was natural.	7	7
(8) Using the application was useful.	6.5	6
(9) I would like to use the application again.	5	5
(10) The application aided my learning.	6	6
(11) Altogether, I am satisfied with the application.	6	6
(12) The support glossary provided by the application was useful.	6	5
(13) The support glossary provided by the application was annoying.	1	1
(14) The support glossary provided by the application helped me to understand the context.	5	5
(15) The support glossary provided by the application helped me to identify errors in my thinking.	4	4
(16) The support glossary provided by the application helped me to understand relationships between entities.	5	5
(17) The support glossary provided by the application helped me learn.	5	5

## References

[1] Murray M. C., Perez J. (2011). E-Textbooks are coming: are we ready?. *Issues in Informing Science and Information Technology*.

[2] Rockinson- Szapkiw A. J., Courduff J., Carter K., Bennett D. (2013). Electronic versus traditional print textbooks: a comparison study on the influence of university students' learning. *Computers & Education*.

[3] Mikkilä-Erdmann M., Södervik I., Raisamo R. E-textbook as a tool for promoting conceptual learning in science—looking for novel design and empirical evidence.

[4] Saarinen S., Hietala P., Mikkilä-Erdmann M., Mäkiaho P., Poranen T., Turunen M. Improving e-textbooks: effects of concept maps.

[5] Novak J. D., Canas A. L. (2006). The theory underlying concept maps and how to construct and use them. *Technical Report IHMC CmapTools*.

[6] Puntambekar S., Stylianou A., Goldstein J. (2007). Comparing classroom enactments of an inquiry curriculum: lessons learned from two teachers. *Journal of the Learning Sciences*.

[7] Hay D., Kinchin I., Lygo-Baker S. (2008). Making learning visible: the role of concept mapping in higher education. *Studies in Higher Education*.

[8] Shaw R.-S. (2010). A study of learning performance of e-learning materials design with knowledge maps. *Computers & Education*.

[9] Hay D. B., Kehoe C., Miquel M. E. (2008). Measuring the quality of e-learning. *British Journal of Educational Technology*.

[10] Broughton S. H., Sinatra G. M., Reynolds R. E. (2010). The nature of the refutation text effect: an investigation of attention allocation. *The Journal of Educational Research*.

[11] Vilppu H., Mikkilä-Erdmann M., Vilppu H., Ahopelto I. (2013). The role of regulation strategies in understanding science text among students. *Scandinavian Journal of Educational Research*.

[12] Mikkilä-Erdmann M. (2001). Improving conceptual change concerning photosynthesis through text design. *Learning and Instruction*.

[13] Vassiliou M., Rowley J. (2008). Progressing the definition of ‘E-book’. *Library Hi Tech*.

[14] Tanhua-Piiroinen E., Pystynen J., Raisamo R. Haptic applications as physics teaching tools.

[15] Raisamo R., Wallden S., Suhonen K., Cruz-Cunha M. M. (2012). Design and evaluation of Tamhattan: a multimodal game promoting awareness of health in a social and positive way. *Handbook of Research on Serious Games as Educational, Business and Research Tools*.

[16] Villalon J., Calvo R., Montenegro R. Analysis of a gold standard for concept map mining—how humans summarize text using concept maps.

[17] Chang K.-E., Sung Y.-T., Chen I.-D. (2002). The effect of concept mapping to enhance text comprehension and summarization. *The Journal of Experimental Education*.

[18] Hauser S., Nückles M., Renkl A. Supporting concept mapping for learning from text.

[19] Nesbit J. C., Adesope O. O. (2006). Learning with concept and knowledge maps: a meta-analysis. *Review of Educational Research*.

[20] Hwang G.-J., Yang L.-H., Wang S.-Y. (2013). A concept map-embedded educational computer game for improving students' learning performance in natural science courses. *Computers & Education*.

[21] Ahopelto I., Mikkilä-Erdmann M., Anto E., Penttinen M. (2011). Future elementary school teachers' conceptual change concerning photosynthesis. *Scandinavian Journal of Educational Research*.

[22] Bostock M. D3—Data driven documents. http://www.d3js.org/.

[23] Mikkilä-Erdmann M., Penttinen M., Anto E., Olkinuora E., Ifenthaler P., Pirnay-Dummer P., Spector J. M. (2008). Constructing mental models during learning from science text. *Understanding Models for Learning and Instruction: Essays in Honor of Norbert M. Seel*.

[24] Kinchin I. M. (2000). Concept mapping in biology. *Journal of Biological Education*.

[25] Turunen M., Hakulinen J., Melto A., Heimonen T., Laivo T., Hella J. SUXES—User experience evaluation method for spoken and multimodal interaction.

[26] Bateman S., Gutwin C., Osgood N., McCalla G. Interactive usability instrumentation.

[27] Fisher C., Sanderson P. (1996). Exploratory sequential data analysis: exploring continuous observational data. *Interactions*.

[28] Kim J. H., Gunn D. V., Schuh E., Phillips B., Pagulayan R. J., Wixon D. Tracking realtime user experience (TRUE): a comprehensive instrumentation solution for complex systems.

[29] Renaud K., Gray P. Making sense of low-level usage data to understand user activities.

[30] Hilbert D. M., Redmiles D. F. (2000). Extracting usability information from user interface events. *ACM Computing Surveys*.

[31] Church K., Cherubini M. Evaluating mobile user experience in-the-wild: prototypes, playgrounds and contextual experience sampling.

[32] Akers D., Jeffries R., Simpson M., Winograd T. (2012). Backtracking events as indicators of usability problems in creation-oriented application. *ACM Transactions on Computer-Human Interaction*.

[33] Harbich S., Hassenzahl M. (2011). Using behavioral patterns to assess the interaction of users and product. *International Journal of Human-Computer Studies*.

[34] Kort J., Steen M. G. D., de Poot H., ter Hofte H., Mulder I. Studying usage of complex applications.

[35] Fern X., Komireddy C., Grigoreanu V., Burnett M. (2010). Mining problem-solving strategies from HCI data. *ACM Transactions on Computer-Human Interaction*.

[36] Mankowski W. C., Bogunovich P., Shokoufandeh A., Slvucci D. D. Finding canonical behaviors in user protocols.

[37] Moura D., Seif el-Nasr M., Shaw C. D. Visualizing and understanding players' behavior in video games: discovering patterns and supporting aggregation and comparison.

[38] Wallner G., Kriglstein S. A spatiotemporal visualization approach for the analysis of gameplay data.

[39] Hurst A., Hudson S. E., Mankoff J. Dynamic detection of novice vs. skilled use without a task model.

[40] Huang J., Zimmermann T., Nagapan N., Harrison C., Phillips B. C. Mastering the art of war: how patterns of gameplay influence skill in Halo.

[41] R Core Team (2011). *R: A Language and Environment for Statistical Computing*.

[42] Gabadinho A., Ritschard G., Müller N. S., Studer M. (2011). Analyzing and visualizing state sequences in R with TraMineR. *Journal of Statistical Software*.

[43] Maechler M., Rousseeuw P., Struyf A., Hubert M., Hornik K. Cluster: Cluster analysis basics and extensions.

[44] Ward J. H. (1963). Hierarchical grouping to optimize an objective function. *Journal of the American Statistical Association*.

